# Convolutional Blur Attention Network for Cell Nuclei Segmentation

**DOI:** 10.3390/s22041586

**Published:** 2022-02-18

**Authors:** Phuong Thi Le, Tuan Pham, Yi-Chiung Hsu, Jia-Ching Wang

**Affiliations:** 1 Department of Biomedical Sciences and Engineering, National Central University, Taoyuan 320317, Taiwan; lethiphuong@g.ncu.edu.tw; 2 Faculty of Digital Technology, University of Technology and Education-The University of Danang, Danang 550000, Vietnam; ptuan@ute.udn.vn; 3 Department of Computer Science and Information Engineering, National Central University, Taoyuan 320317, Taiwan

**Keywords:** cell nuclei, convolutional neural network, deep learning, nucleus segmentation

## Abstract

Accurately segmented nuclei are important, not only for cancer classification, but also for predicting treatment effectiveness and other biomedical applications. However, the diversity of cell types, various external factors, and illumination conditions make nucleus segmentation a challenging task. In this work, we present a new deep learning-based method for cell nucleus segmentation. The proposed convolutional blur attention (CBA) network consists of downsampling and upsampling procedures. A blur attention module and a blur pooling operation are used to retain the feature salience and avoid noise generation in the downsampling procedure. A pyramid blur pooling (PBP) module is proposed to capture the multi-scale information in the upsampling procedure. The superiority of the proposed method has been compared with a few prior segmentation models, namely U-Net, ENet, SegNet, LinkNet, and Mask RCNN on the 2018 Data Science Bowl (DSB) challenge dataset and the multi-organ nucleus segmentation (MoNuSeg) at MICCAI 2018. The Dice similarity coefficient and some evaluation matrices, such as F1 score, recall, precision, and average Jaccard index (*AJI*) were used to evaluate the segmentation efficiency of these models. Overall, the proposal method in this paper has the best performance, the *AJI* indicator on the DSB dataset and MoNuSeg is 0.8429, 0.7985, respectively.

## 1. Introduction

Artificial intelligence (AI) enables computers to perform human tasks. The development of AI can be observed in two topics, namely machine learning and deep learning. There are currently many AI applications, including housework robots, autonomous cars, chatbots, etc. The success of an AI application relies on the availability of the related data. In particular, the cell nuclei biomedical datasets help the researchers and doctors in disease prognosis as well as facilitate drug development and medical treatment.

The cell nuclei in the human body contain the DNA or the genetic codes, which need to be identified in the first step of the analysis. Cell analysis enables researchers to determine whether or not the cells will react to certain drug treatments. By analyzing the cells in drug development, the researchers are able to suggest new treatment methods and improve the patient’s health. Due to the demands for automatic cell nuclei analysis, many research works have been conducted on related topics. For instance, in actin-labeled fluorescence microscopy images, cell nuclei classification provides information regarding malignant alterations and can be applied to enhance diagnostic markers to delimit the relative position, as well as determine the normal and cancerous breast cells [1]. By classifying the cell nuclei, the authors in [2,3] have successfully classified the cell atlases for the tissue and Hep-2 cells. The cell nuclei have also been analyzed by applying nucleus detection on high-resolution, histopathology, breast cancer images to categorize the subjects in the images into nuclear or non-nuclear [4]. In other aspects, nucleus detection has been implemented for different datasets by manually marking the subjects in each image [5,6,7]. More recently, nucleus detection and classification have been combined simultaneously for a typical histopathology image dataset [8].

Although the detection and classification of cell nuclei are important in learning image features and supporting disease diagnoses, these tasks have several drawbacks. As the boundaries of the subjects are not recognized exactly, only the relative outlines of the subjects are drawn, which reduces the applicability. Therefore, nucleus segmentation is essential in determining the positions and boundaries of the nuclei. Previous studies have also investigated nucleus segmentation. For example, the watershed algorithm [9], K-means clustering [10], and Otsu’s algorithm [11] are three well-known algorithms for nucleus segmentation. Unfortunately, these algorithms are less accurate for nucleus segmentation because they are sensitive to different parameter settings. They may have a particular set of parameter values that corresponds to specific nuclei structures and image extraction conditions. These drawbacks decrease the applicability of the mentioned classical nucleus segmentation algorithms in real situations.

To enhance the segmentation accuracy, a deep learning model is used to automatically segment the cell nucleus. For instance, Ronneberger designed the U-Net model for biomedical image segmentation including nucleus segmentation [12]. The architecture contains a symmetric expanding path and a contracting path to allow precise localization and context capture, respectively. Johnson and Jung et al. used the Mask-RCNN model for nucleus segmentation [13,14]. The Mask-RCNN model [15] proposes the possible object regions and the bounding box in the first step, where a mask of the object is drawn at the pixel level. This model has good performance in nucleus segmentation with respect to both the accuracy and processing speed. Hollandi et al. used image style transfer to increase the number of nuclei training images [16]. The Mask-RCNN model was trained on augmented training data. To refine the segmentation result in pixel-level accuracy, the edges of the detected nuclei were corrected using the U-Net model with mathematical morphology-based post-processing. Feixiao long proposed an enhanced U-Net model with a modified encoded branch for nucleus segmentation [17], which achieved higher precision than variant models of the U-Net. The goal of this work, motivated by the success of deep learning, is to provide more accurate nucleus segmentation for doctors, patients, and researchers in disease prognosis. In this paper, we present a convolutional blur attention (CBA) network for automatic nucleus segmentation. This network is able to assign deterministic labels to the pixels through the features of input images. It has been found that our model has the highest performance when evaluated by the intersection over union (IoU) measure.

The rest of the paper is organized as follows. In Section 2, we describe the dataset used in this work and the architecture of the proposed convolutional blur attention network in detail. The experimental results and discussion are given in Section 3. Finally, we provide the conclusions in Section 4.

## 2. Materials and Methods

### 2.1. Dataset

The cell nucleus segmentation dataset used in this study comes from the 2018 Data Science Bowl challenge [18].

**Figure 1:**
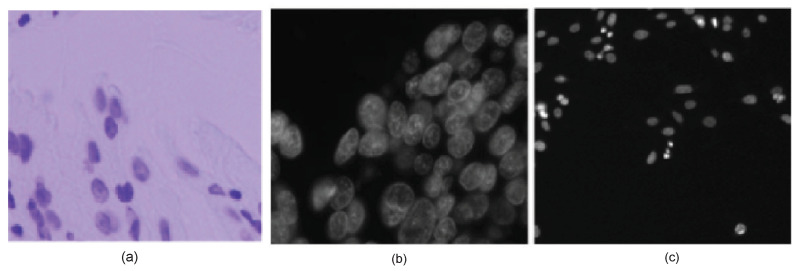
Diversity within the original images: (**a**) heterogeneous illumination. (**b**) Boundaries between the nucleus are ambiguous. (**c**) Microscope magnification and variety of cell types.

The goal of this challenge was to find nuclei in various input images in order to meet the demand for advanced medical discovery. The dataset collected nuclei images from various sources, such as research biologists in hospitals, universities, and biotechs. The nuclei in this dataset were derived from the cell nuclei images of humans, mice, and flies. The dataset included diverse cell types with various external factors, such as illumination, histology stains, or magnifications. For instance, the microscopy images were taken from 15 different biological experiments with a lack of (or insufficient) illumination. Figure 1 illustrates the diverse appearances of the cells in the original images. Figure 1a is an example of heterogeneous illumination. Without contrast adjustment, the boundaries between cells are ambiguous, as shown in Figure 1b, which may cause difficulties in nuclei segmentation. The efficiency of nuclei segmentation is also affected significantly by the microscopy magnification and various cell samples, as shown in Figure 1c.

Secondly, the multi-organ nucleus segmentation (MoNuSeg) at MICCAI 2018 includes digital microscopic tissue images as implemented in this paper [19]. The MoNuSeg contains 30 images on training and 14 images on testing. In truth, 30 images in training data with each size, 1000 × 1000, were extracted from 21,623 annotated nuclei of various organs, such as breast, bladder, stomach, liver, colon, kidney, and prostate. Furthermore, 7223 hand-annotated boundaries of these organs are represented in 14 testing images, each of size 1000 × 1000 pixels. The variety of nuclei images allows for the development of nuclei segmentation techniques that are both robust and generalizable.

### 2.2. Convolutional Blur Attention Network

**Figure 2:**
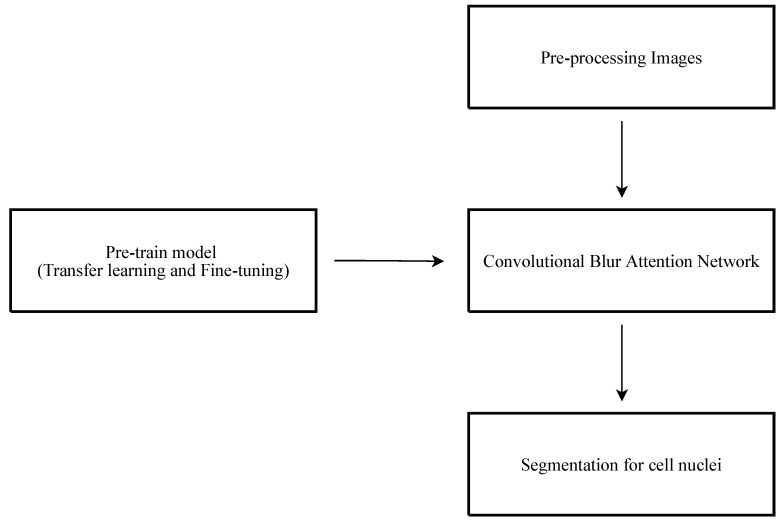
The flowchart of the proposed method.

The flowchart of the proposed method is given in Figure 2. The original images must be used for preprocessing in order to normalize the images and augment the training image data. The proposed CBA network is pre-trained first and fine-tuned to the training image data. The purpose of pre-trained step is to minimize the computational requirement and emphasize the subject. In the preprocessing step, the input images are scaled down to 192 by 192 pixels and converted from RGB to grayscale that retain sufficient quality for background and nucleus segmentation.

**Figure 3:**
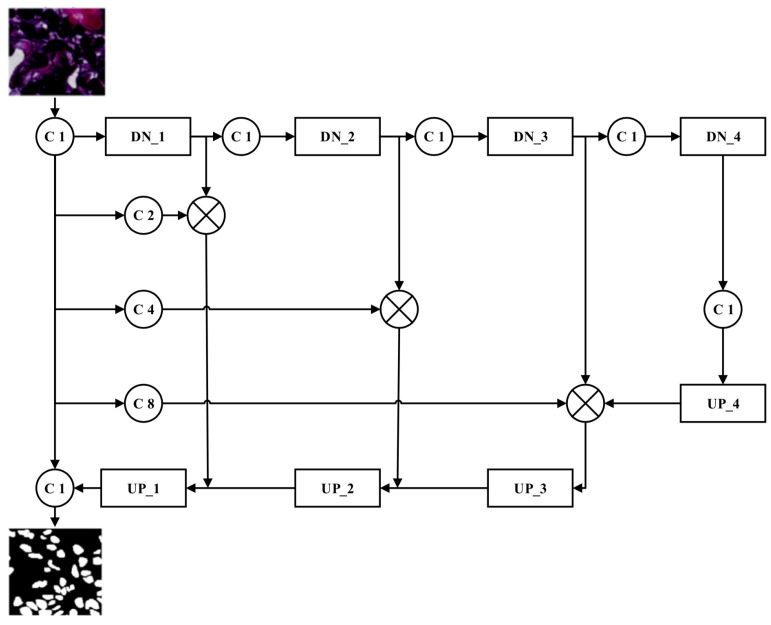
The architecture of convolutional blur attention networks.

**Figure 4:**
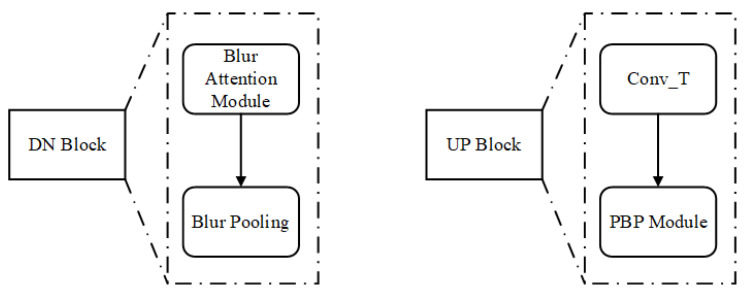
Block diagram of DN block (**left**); block diagram of UP block (**right**).

The architecture of the proposed convolutional blur attention network is shown in Figure 3. Most models using in image segmentation were designed with max-pooling in the downsampling path to reduce image sizes. This step causes the big problems reduce accuracy in image segmentation because of loss features. To overcome this drawback, the downsampling in the proposed method has attention module and blur-pooling to learn more (and closer) features at output. In addition, in the proposal study, there is the pyramid module at the upsampling path to learn features under different viewings. Moreover, to make sure the original features keep to the upsampling, the auxiliary connections were proposed in this study. Moreover, the pre-trained model distributes significantly, to enhance performance in this study. Firstly, downsampling and upsampling are the two main paths in this network. The circle blocks C1, C2, C4, and C8 are convolutions with stride values of 1, 2, 4 and 8, respectively. The DN_1, DN_2, DN_3, and DN_4 represent the downsampling (DN) blocks, as shown in Figure 4 (left). The UP_1, UP_2, UP_3, and UP_4 represent the upsampling (UP) blocks, as shown in Figure 4 (right).

The downsampling stage is essential during the segmentation because it catches and encodes the salient characteristics of the input images into a smaller feature map. In our downsampling stage, a convolution (C1) is first applied to the input image to generate the input image features before entering DN_1.

More interestingly, instead of using a traditional convolution, we designed a blur convolution with a stride value of 1 to be the (C1) convolution block. There are several DN blocks in the downsampling stage. The DN block includes a blur attention module and a blur pooling operation to reduce the image feature dimensions. In truth, downsampling in the pooling layer caused a shift-invariant to be lost. It is still possible to fix shift-invariant by simply analyzing densely extracted features [20,21]. Blur pooling [22] was proposed by Zhang to improve shift-invariant loss. The blur-pooling layer was decomposed into two steps: (1) dense evaluating of the max operator and (2) naïve sub-sampling. The authors proposed adding a low-pass filter between them in effort to eliminate aliasing. With this approach, low-pass filtering augments, rather than replaces max-pooling layers. So anti-aliasing and max-pooling can be combined in a novel way, in that shifts in the input have relatively little impact on output (shift-invariant).

**Figure 5:**
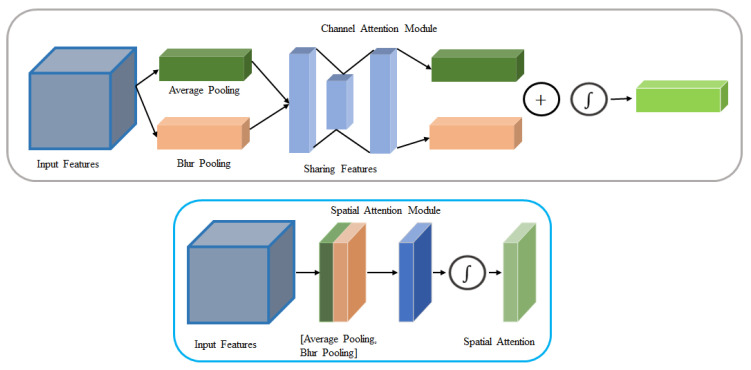
The architecture of the blur attention module.

In the blur attention module, this study enhances the convolutional block attention module [23] by integrating the blur pooling operation. The architecture of the blur attention module is described in Figure 5.

This blur attention module includes the channel blur attention and spatial blur attention units. In the channel blur attention unit, the attention is obtained by the accumulation of spatial information through average pooling and blur pooling. Average pooling provides the spatial statistics [24] and blur pooling learns the object features effectively. The two pooling results are fed into a multi-layer perception and the associated outputs are summed element-wise to generate the channel blur attention. In the spatial blur attention unit, the attention is obtained by accumulating the channel information using average pooling and blur pooling along the channel axis. A convolution layer is used to filter the concatenated pooling results and generate the attention that identifies spatially important regions. The channel blur attention $M_{CB}$ and the spatial blur attention $M_{SB}$ are computed as:

(1) $$M_{CB}=\sigma \left(MLP\left(AveragePooling\left(F\right)\right)+MLP\left(BlurPooling\left(F\right)\right)\right)$$

(2) $$M_{SB}=\sigma (f^{3\times 3}\left(\left[AveragePooling\left(F\right);BlurPooling\left(F\right)\right]\right)$$

(3) $$F_{out}=\left(M_{CB}⨂F\right)⨂M_{SB}$$

where σ, ⨂, *MLP* denote the sigmoid function, element-wise multiplication, and the multi- layer perceptron. *F*, $F_{out}$ and $f^{3\times 3}$ represent input feature map, output, and a convolution operation with filter size of 3 × 3.

In the blur attention module, the input features are multiplied by the channel blur attention and the spatial blur attention. This process enhances the feature extraction with respect to the contents and positions.

In our experiment, reducing the image size by a factor of 4 is sufficient; therefore, the DN block is applied four times in the downsampling stage to reduce the image size from 192 × 192 to 12 × 12. At DN_1, the size of the input image is reduced from 192 × 192 to 96 × 96. Similarly, the image sizes are reduced to 48 × 48, 24 × 24 and 12 × 12, after DN_2, DN_3 and DN_4, respectively. Although the sizes of the image features are diminished in the downsampling stage, the proposed blur attention module contributes significantly to the encoding of beneficial image features.

**Figure 6:**
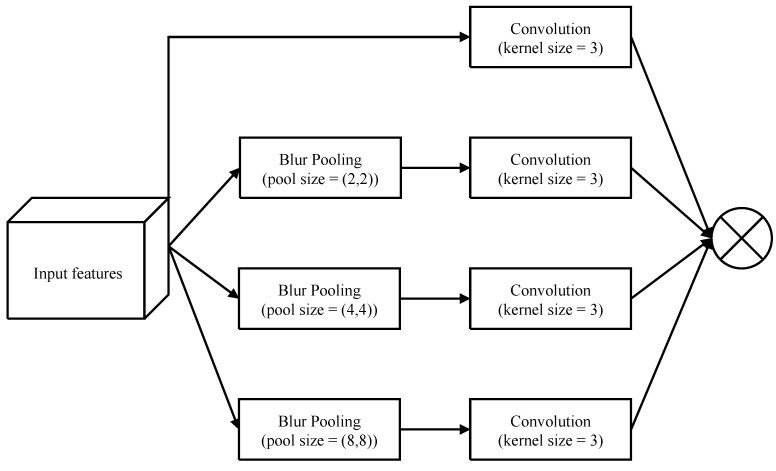
The architecture of the proposed pyramid blur pooling module.

After completing the downsampling processes, the model uses an upsampling process as the decoder. The key function of upsampling is to return the image sizes back to the original dimensions and is achieved through a deconvolution operation using transpose convolution (Conv_T) with a 3 × 3 kernel size and a stride value of 2. Four transpose convolutions are required if downsampling was performed four times. After trials with different datasets, it was found that a significant amount of information is lost during the downsampling and upsampling processes. To avoid information loss, we propose to extract information from the C1-convoluted input image through convolutions with different strides (C2, C4, and C8). Rather than using blur pooling, this process reduces the input image sizes to 96, 48, and 24 through convolutions with stride values of 2, 4, and 8, respectively. The features obtained from this strategy are concatenated with the features from the DN block at the same size to provide more abundant features at the upsampling stage. The other advantage of this network is the addition of the proposed pyramid blur pooling (PBP) module to each UP block to capture and disclose neighboring features with high correlations to the context information before the change in image size. The architecture of the proposed pyramid blur pooling module is shown in Figure 6. The PBP module enhances the traditional pyramid pooling module (PPM) [25] by performing the blur convolution. The blur convolution is designed by integrating blur pooling and convolution. The atrous convolution layers are applied in parallel to capture various information simultaneously. Four filters with dimensions of 1 × 1, 2 × 2, 4 × 4, and 8 × 8 are used to explore the multi-scale features. Finally, these filter outputs are upsampled and concatenated together.

## 3. Results and Discussion

### 3.1. Pre-Processing and Evaluation Criterion

In our experiments, 670 images from the Data Science Bowl challenge dataset described in Section 2.1 were used. Thus, 80% of the images were applied for training and 20% for testing. The numbers of training and testing images were 536 and 134, respectively. To be more specific, we believe that increasing the number of input images through random rotation and splitting will improve training efficiency. As a result, 536 images with different sizes transferred to 2372 images with 192 × 192 sizes. For the MoNuSeg dataset, to ensure sufficient data in the train and test processing, we extracted each original image of 1000 × 1000 to sub-patches of 250 × 250 sizes, resulting in each original image being divided into 16 smaller ones. After completing the step, the number of images increased from 30 to 480 images on the training set and from 14 to 224 images on the testing as shown in Table 1 below.

Moreover, in order to enhance quality in training images and increase the quantity of input images, we used train data generation for the training datasets. During the training process, the model will never see twice the same image. This is also important in the testing process because it prevents over-fitting problems and helps the model generalize better.

Besides, there is increasing interest in discrete wavelet transforms (DWTs) in order to reduce image noise (denoising), store, and retrieve images. Wavelet denoising is effective at many applications [26]. There are a several wavelet types, such as both biorthogonal and coiflets with Daubechies, Haar, Meyer, reverse biorthogonal, and symlet. In this paper, we applied biorthogonal wavelet to implement the low pass filter.

To evaluate the nucleus segmentation performance, the Dice similarity coefficient (*DSC*) [27] was used to measure the spatial overlap between two sets. The *DSC* is defined as:

(4) $$DSC=\frac{2*(A\cap B)}{A+B}$$

*A* and *B* are two target regions. ∩ is defined as the intersection between the two sets. For an object level evaluation matrix, we used the aggregated Jaccard index (*AJI*) to evaluate [28]. The *AJI* is essentially an extension of the Jaccard index.

(5) $$AJI=\frac{\sum_{i=1}^{L}|G_{i}\cap P_{i}|}{\sum_{i=1}^{L}|G_{i}\cap P_{i}|+\sum_{i\in rest}|P_{i}|}$$

where $G_{i}$ is made up of pixels from the whole ground true of the *i*th nucleus with *L* nuclei. $P_{i}$ is the predicted nucleus that has the maximum Jaccard index with $G_{i}$. The term “*rest*” refers to the sum of $P_{i}$ that has no match. The *AJI* is the ratio of the common region of matched elements to the segmented results. Any inaccuracy in segmentation, whether over- or undersegmentation, will lead to a decrease in *AJI*. For pixel-level evaluation metrics, $F_{1}$ score, precision, and recall were used in this study to evaluate [29]. The calculation of $F_{1}$ score, precision, and recall are described below:

(6) $$F_{1}=\frac{2*Precision*Recall}{Precision+Recall}$$

(7) $$Precision=\frac{TP}{TP+FP}$$

(8) $$Recall=\frac{TP}{FN+TP}$$

where *TP* is true positive, *FP* is false positive, *FN* is false negative.

To obtain a better trained model, this study also utilizes the pre-training and fine-tuning techniques whose advantages have been demonstrated in many research works [15,29,30]. This process is useful in preventing overfitting for a small training dataset.

### 3.2. Experiment Results for MoNuSeg Dataset

**Figure 7:**
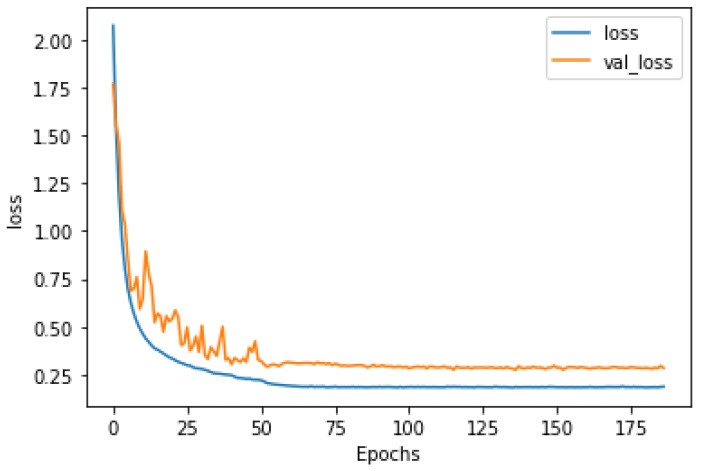
Loss curve for the MoNuSeg dataset.

From Figure 7, we could see the training loss as well as the validation loss reduce independently after each epoch. This reveals the stabilization and efficiency during the training and validation without overfitting.

Based on the evaluation metrics, we consider some previous methods, such as U-Net [12], LinkNet [31], SegNet [32], and ENet [33] as baseline models to compare the efficiency with the proposed method. This dataset splits in training and testing. Table 2 depicts the evaluation results of the baseline models as well as the proposal for MoNuSeg. According to Table 2, the performance of U-Net and LinkNet are quite similar, and the average of the Dice coefficient, *AJI* scores of U-Net and LinkNet are lower than SegNet and ENet. Moreover, all evaluation indicators of ENet are higher than U-Net, LinkNet, and SegNet. On the other hand, the proposal method has a better performance than the other under the evaluation of the Dice coefficient, F1, precision, *AJI*. It is clear that U-Net has the lowest performance in the four baseline models, in contrast, ENet has the highest performance. However, the proposed model reached the highest score with the *AJI* of 0.7925 versus U-Net (0.3605), versus LinkNet (0.3651), versus SegNet (0.4003), and versus ENet (0.51).

**Figure 8:**
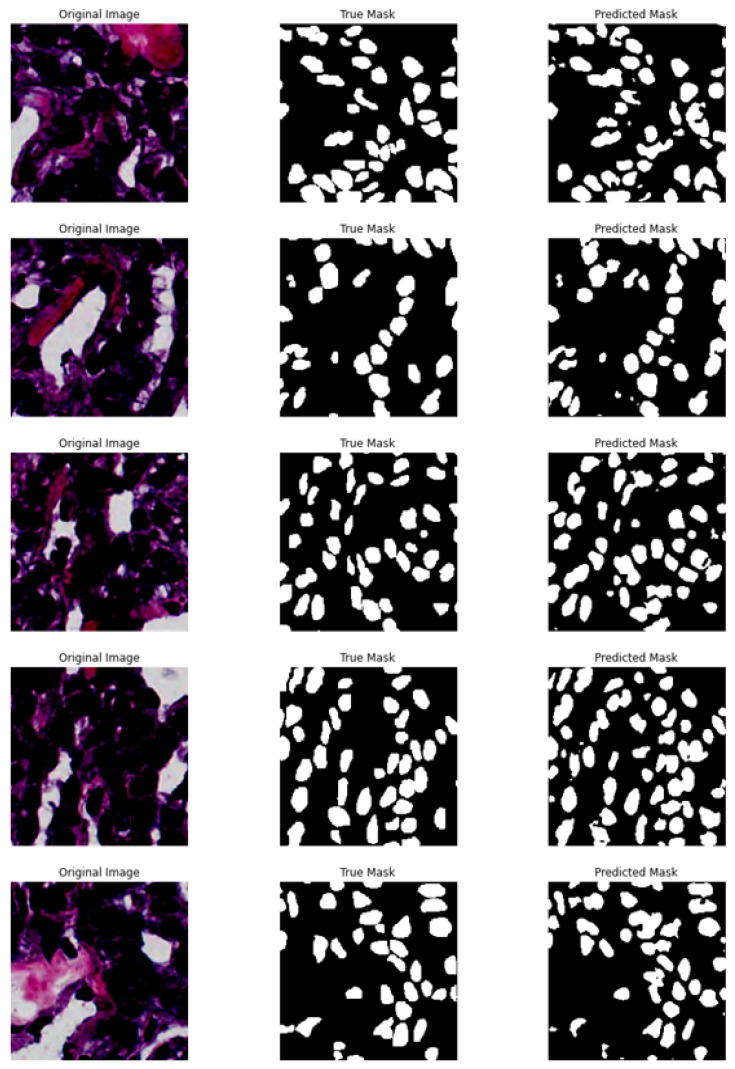
From left to right: the original images, the ground-truth segmentation, the predicted segmentation for the MoNuSeg dataset.

The segmentation visualization of the proposed model is shown in Figure 8 below. Overall, the masks predicted by the proposal are pretty much identical, despite the fact that some nuclei were not predicted precisely. According to the complicated image construction, we could not segment nuclei exactly by “normal eyes” for the MoNuSeg dataset; therefore, the computer skipped some nuclei, which is acceptable. Furthermore, the original image quantity (in total, 44 images) is really small. This strongly affects the training and evaluation effectiveness of the model.

### 3.3. Experiment Results for DSB Dataset

**Figure 9:**
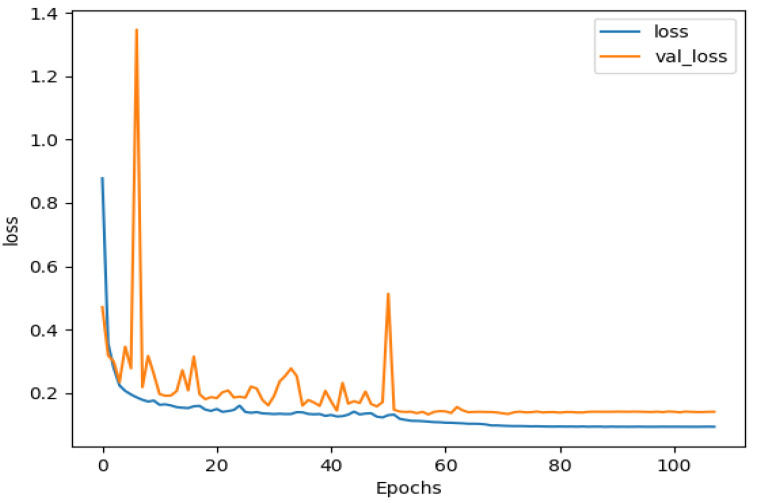
Loss curve for the DSB dataset.

Figure 9 depicts the loss curve in the training and validation processing for the DSB dataset. During training, the loss values continuously decrease after each epoch. For validation loss, the value reduces unevenly in the first epochs, but it becomes smooth in the last epochs.

**Figure 10:**
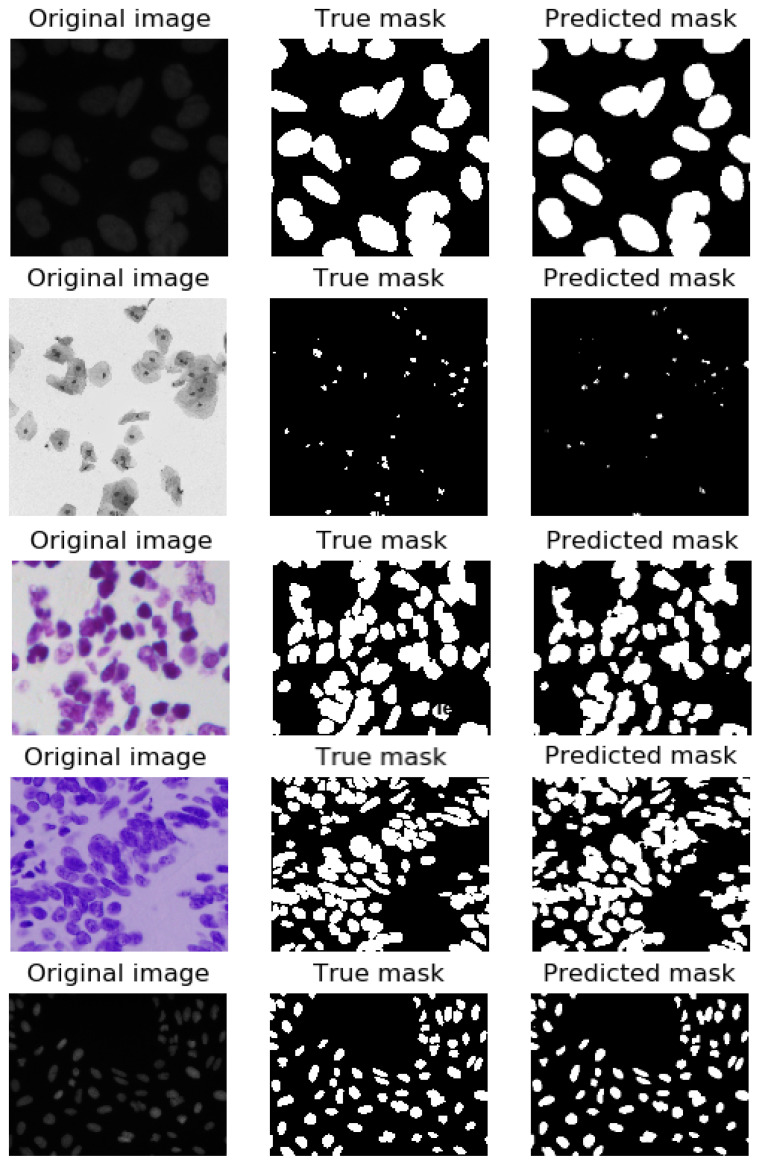
From left to right: the original images, the ground-truth segmentation, the predicted segmentation for the DSB dataset.

The nucleus segmentation performances of the four baseline models, Mask RCNN with ResNet-50 [13], Mask RCNN with ResNet-101 [13], and the proposal are also compared for the Data Science Bowl challenge dataset. Table 3 presents the performance comparisons as given by the dice coefficient, F1, recall, precision, and *AJI*. The segmentation visualization of the proposed model is shown in Figure 10 below. The evaluated indicators of U-Net are the lowest. The precision indicator of SegNet (0.945) is higher than ENet (0.9264). In contrast, the other scores of the ENet are better than SegNet as well as LinkNet and U-Net. The U-Net model yields the *AJI* of 0.4521. The *AJI* values yielded by the Mask RCNN with ResNet-50 and the Mask RCNN with ResNet-101 are 0.7106 and 0.74, respectively. The proposed model yields the highest *AJI* of 0.8429. Furthermore, the proposal achieves the highest score for all indicators out of all of these methods.

### 3.4. Discussion

**Figure 11:**
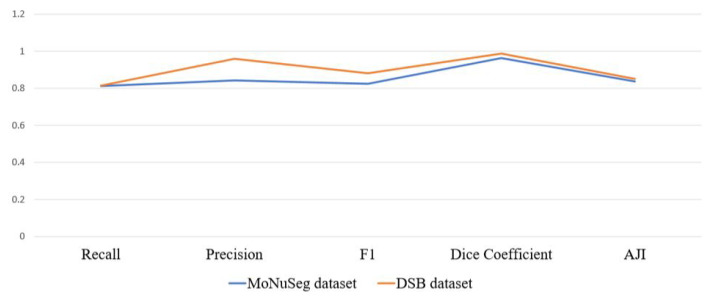
The evaluated indicators for the MoNuSeg and DSB datasets were performed by the proposal.

Although the MoNuSeg and DSB datasets are different in quantity as well as quality, these datasets were trained and evaluated in the same process. We could see the main trend through Figure 11 that the dice coefficient achieved the highest scores. Furthermore, the precision indicator is higher than recall. It means that the correctly segmented nuclei pixels to the total number of pixels are higher compared to the total number of nuclei pixels per an image. The network returned the *AJI* indicator for the DSB dataset as higher than for the MoNuSeg dataset. This emphasizes once more how important the original data are for nuclei segmentation. In reality, the DSB dataset contains 670 images, whereas the MoNuSeg dataset contains 44 images. In addition, there are many difficulties in the datasets: different color contrasts and the arrangements of nuclei in different histopathology images. In the future, besides thinking about creating more nuclei dataset, we will focus on the pre-processing.

**Figure 12:**
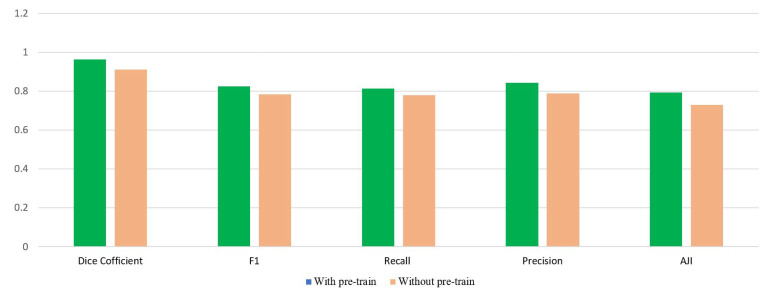
The performance comparison of the CBA model with and without the pre-trained model in the MoNuSeg dataset.

**Figure 13:**
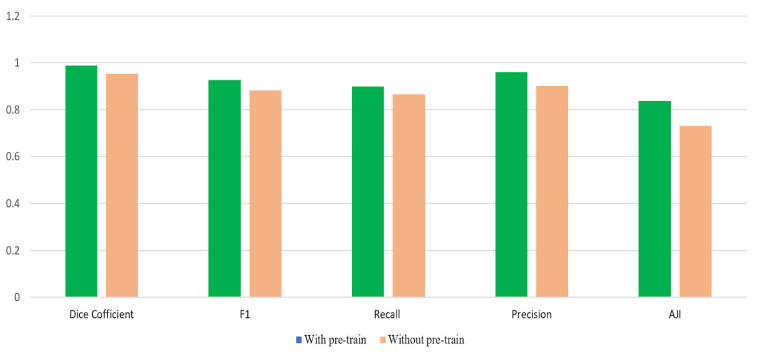
The performance comparison of the CBA model with and without the pre-trained model in the DSB dataset.

As for whether or not to apply the pre-train model, the experimental comparison is shown in Figure 12, and Figure 13 for the MoNuSeg and the DSB dataset respectively. For Figure 12, the nuclei segmentation efficiency with the pre-trained model in the MoNuSeg dataset is higher than without the pre-trained model under the same conditions. Similarly, It could be seen from Figure 13 that the performance of the model in the DSB dataset with the pre-trained model is slightly better than without the pre-trained model. Overall, the pre-trained paradigm boosts the model performance, although the increase is minor due to the limit on the amount of input images.

Actually, the proposed model not only achieves impressive results, it is also useful to train via a personal computer, without a server or a big computer, because of less parameters compared to the others, as described in Table 4.

The visualization results of the U-Net, LinkNet, SegNet, ENet, and the proposal model are shown in Appendix A. The left to the right of the column present original images, true mask, and predicted mask by the proposal, U-Net, LinkNet, SegNet, and ENet. The concise structure makes the training process easy, but the ability of the independent nuclei segmentation is weak, resulting in under segmentation of cluster nuclei. Moreover, the performance of the LinkNet model does not overcome the challenge in pathological images with numerous small-area nuclei in pathological images. The U-Net and LinkNet models zone out the nuclei in general, but these models do not exactly segment the area of each nucleus. The performances of the SegNet and ENet network “improve” compared to the U-Net and LinkNet; however, these models produce more noise in segmented images. Overall, we can see that the segmented results of our model (in the third column) are very close to the true mask (in the second column).

## 4. Conclusions

In this paper, we proposed use of the CBA network to perform cell nuclei segmentation without manual intervention. This approach is able to overcome difficulties, such as the overfitting problem caused by a limited number of input images. In addition, the drawbacks related to the variety of cell image styles, small subjects, and hair noise in the input images were resolved using blur pooling in conjunction with the attention and pyramid modules at the downsampling and upsampling paths, which eliminated shift-variance loss and improved segmented efficiency. Moreover, the pre-processing step with the low-pass filter technique and the pre-trained model also significantly contributed toward enhancing features in training. In the experiments, we compared the segmentation results of the CBA network, U-Net, ENet, SegNet, LinkNet, Mask-RCNN, and ResNet models on two datasets, namely MoNuSeg and DSB. This network performs better than the other models, according to the evaluating indicators as well as visualizing images. The average *AJI* indicator of the proposed network is the highest, with 79.85% for the DSB dataset and 84.29% for the MoNuSeg dataset. Furthermore, the number of parameters in our model “reduces triple” in Mask-RCNN, which results in negligible computational overhead when running our model, and provides superior performance for nuclei segmentations.

In the future, we will explore our network using biomedical images from other sources. Furthermore, developing more effective post-processing methods to address the issue of cell overlap is important. In addition, self-supervised learning is worthy of attention, and could apply to less input images, such as biomedical datasets.

## Figures and Tables

**Table 1:** The quantity of training and testing images changed pre and post the preprocessing step.

	MoNuSeg Dataset		DSB Dataset	
Pre-processing	before	after	before	after
Training/Testing	30/14	480/224	536/134	2372/134

**Table 2:** Quantitative comparison of different methods applied to MoNuSeg dataset.

Methods	Evaluated Indicators				
	Dice Coefficient	F1	Recall	Precision	*AJI*
U-Net [12]	0.8092	0.65	0.8275	0.5358	0.3605
LinkNet [29]	0.8292	0.6511	0.8386	0.5322	0.3651
SegNet [30]	0.8885	0.6771	0.876	0.5518	0.4003
ENet [31]	0.9020	0.6769	0.9208	0.5352	0.51
Mask RCNN with ResNet-50 [13]	0.9181	0.7247	0.7338	0.7158	0.6217
Mask RCNN with ResNet-101 [13]	0.9394	0.7519	0.7522	0.7516	0.6551
The proposed model without blur-pooling	0.9427	0.7640	0.7579	0.7701	0.6805
Ours	0.9634	0.8247	0.8125	0.8429	0.7985

**Table 3:** Quantitative comparison of different methods applied to the DSB dataset.

Methods	Evaluated Indicators				
	Dice Coefficient	F1	Recall	Precision	*AJI*
U-Net [12]	0.8524	0.5394	0.4787	0.6177	0.4521
LinkNet [29]	0.9203	0.7846	0.7539	0.8181	0.528
SegNet [30]	0.9447	0.8265	0.7344	0.945	0.694
ENet [31]	0.9462	0.8576	0.7982	0.9265	0.724
Mask RCNN with ResNet-50 [13]	0.9489	0.8453	0.7576	0.956	0.7106
Mask RCNN with ResNet-101 [13]	0.9502	0.8693	0.8052	0.9444	0.74
The proposed model without blur-pooling	0.9572	0.9096	0.8735	0.9488	0.7895
Ours	0.9879	0.9282	0.8989	0.9596	0.8429

**Table 4:** The number of parameters in the models.

Model	Parameters (millions)
U-Net	2.0
LinkNet	11.5
SegNet	29.5
ENet	0.37
Mask RCNN	64.1
Our proposed model	5.8

**Figure A1:**
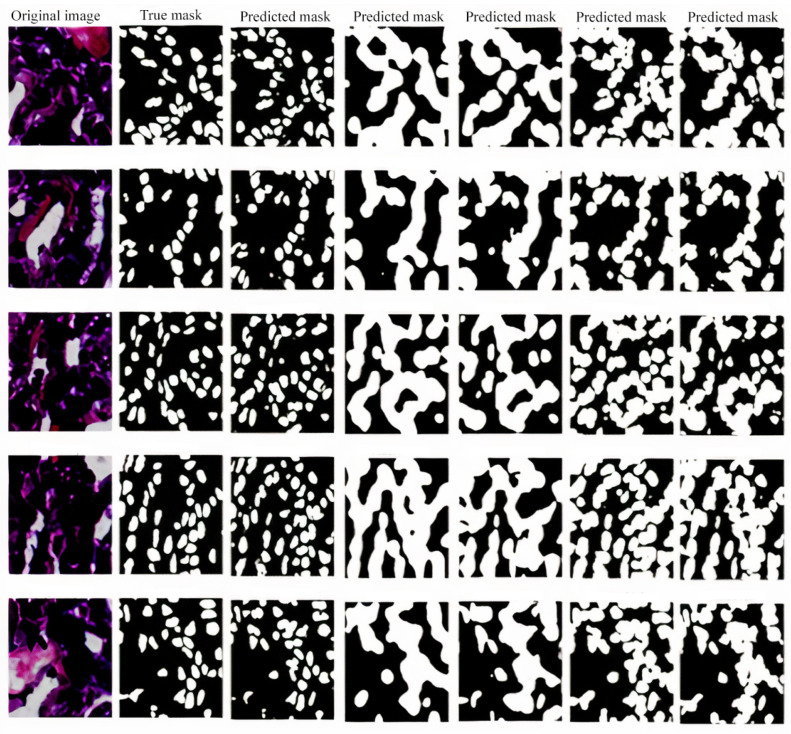
From left to right: The original image, the true mask, the predicted mask by the proposal, U-Net, LinkNet, SegNet, and ENet (see section for detailed analysis).

## Data Availability

The data presented in this study are openly available in the 2018 Data Science Bowl challenge at https://bbbc.broadinstitute.org/BBBC038 (accessed on 18 January 2018) [18], and in a multi-organ nucleus segmentation challenge, at https://monuseg.grand-challenge.org/ (accessed on 20 October 2018) [19].
